# Hysteroscopic versus histopathological agreement in the diagnosis of chronic endometritis: results from a retrospective observational study

**DOI:** 10.1007/s00404-023-07163-w

**Published:** 2023-09-22

**Authors:** Belén Almoguera Pérez-Cejuela, Salvatore Giovanni Vitale, Tirso Pérez-Medina, Mar Rios-Vallejo, Luigi Della Corte, Ana Royuela Vicente, Stefano Angioni, Laura Calles-Sastre

**Affiliations:** 1 Gynecology and Obstetrics Department, Puerta de Hierro Hospital, Autónoma University of Madrid, 28223 Majadahonda, Madrid Spain; 2 Division of Gynecology and Obstetrics, Department of Surgical Sciences, University of Cagliari, 09124 Cagliari, Italy; 3 Department of Neuroscience, Reproductive Sciences and Dentistry, School of Medicine, University of Naples Federico II, 80131 Naples, Italy

**Keywords:** Chronic endometritis, Hysteroscopy, Histology, Infertility, Inflammation

## Abstract

**Purpose:**

To evaluate the agreement rate between hysteroscopy and pathological examination in case of chronic endometritis.

**Methods:**

A retrospective observational study carried out at Gynecology and Obstetrics Department, Puerta de Hierro Hospital, Autónoma University of Madrid, Spain, from January 2021 to June 2022 was performed by obtaining data from 115 medical records of women who underwent office hysteroscopies that was compared with the findings of final histological examination of endometrial biopsy. Cohen's kappa index was used to evaluate this agreement rate. In addition, sensitivity, specificity, positive and negative predictive value and diagnostic accuracy were obtained.

**Results:**

The agreement between hysteroscopic findings and histological examination showed a modest result with a Cohen’s kappa index of 34%. In addition, we obtained a specificity of 70% and a sensitivity of 64%. The positive and negative predictive value were 60.8% and 73.4%, respectively. An excellent agreement rate (100%) between histological and hysteroscopic results was observed in presence of hyperemia and micropolyps.

**Conclusion:**

Although the sample size is not as large as that of other studies published so far, the first glance of our experience is that hysteroscopic signs are not yet sufficient to make an accurate diagnosis of chronic endometritis, thus requiring a histopathological confirmation to make it.

## What does this study add to the clinical work

**Table:** 

Hysteroscopic signs are not yet sufficient to make an accurate diagnosis of chronic endometritis. Histopathological confirmation of chronic endometritis is always necessary for the final diagnosis	

## Introduction

Chronic endometritis (CE) is currently one of the most discussed topic in the field of reproduction. It is characterized by the presence of stromal edema, homogeneous or, more frequently, non-homogeneous endometrial thickening and focal or diffuse periglandular hyperemia with plasmatic cells penetrating the endometrial stroma [1, 2].

Chronic endometritis is rarely suspected and diagnosed, because it is often clinically silent: nevertheless, pelvic pain, dysfunctional uterine bleeding, dyspareunia, and leukorrhea are not so infrequent symptoms [3]. This condition has been linked to unexplained recurrent miscarriage or repeated implantation failure, but estimating its prevalence continues to be challenging: indeed, in the scientific community, more researchers are only partially convinced that it is a real cause of female infertility [4].

Standardized diagnostic criteria for the diagnosis of CE are still lacking, and the increasing rate of CE cases in women with infertility or recurrent miscarriage, even 60% in some case studies [5–7], has turned this into a topic of constant and relevant study. Gradually, the diagnostic criteria seem to be approaching some form of unification, in order to reduce interobserver variability, as, for example, Liu et al. proposed a hysteroscopic morphologic scoring system with high sensitivity and specificity for CE [8].

The aim of this retrospective observational study was to evaluate the agreement rate between hysteroscopic signs and pathological examination in case of CE, also reviewing the most valuable data published so far in the literature.

## Materials and methods

The medical records of 115 consecutive patients who underwent hysteroscopy at the Gynecology and Obstetrics Department, Puerta de Hierro Hospital, Autónoma University of Madrid, Spain, from January 2021 to June 2022 were reviewed. All procedures performed in the study were in accordance with the ethical standards of the institutional and/or national research committee and with the 1964 Helsinki Declaration and its later amendments or comparable ethical standards. The data presented are an amalgamation of hospital-registered audits of current clinical practice. All patients gave their written informed consent before performing the procedure.

In detail, selected hysteroscopies were from young women (37.09 ± 4.69 years old) with no uterine malformations at ultrasound examination, but with a known history of infertility or recurrent miscarriage. More than three quarters of patients had no previous uterine surgery, by laparoscopy or laparotomy, or hysteroscopic treatments (Table 1). Hysteroscopies were performed during the follicular phase of the menstrual cycle (between 3 and 7 days after menstruation) with a vaginoscopic approach and using saline solution as the distending medium (pressures between 65 and 75 mm Hg). The instruments used were a *Storz Bettochi Hysteroscope* (Outer Sheath 4.2 mm and Inner Sheath 3.6 mm) with flexible biopsy forceps, introduced trough a 5 Fr operative channel of the hysteroscope, and a fiber optic light cable. In detail, the forceps were placed, with its jaws opened, against the endometrium and pushed into the tissue for 0.5 to 1 cm. Once a large portion of mucosa was tangentially detached, the jaws were closed and the entire hysteroscope was removed from the uterine cavity, without pulling the tip of the instrument back into the channel. This method allowed us to collect a larger amount of tissue [9].

**Table 1:** Patient’s characteristics: data are expressed as mean and standard deviation

Age (years)	37.09 ± 4.69
Comorbidities	
Hypertension	8/115 (7%)
Diabetes	5/115 (4.4%)
Hypothyroidism	15/115 (13%)
None	87/115 (75.6%)
D&C	0.64 ± 0.87
PID	0.03 ± 0.18
Pregnancy	0.67 ± 0.68
Miscarriages	1.72 ± 1.24
Previous uterine surgery	
Laparoscopic myomectomy	12/115 (10.4%)
Laparotomic myomectomy	2/115 (1.7%)
None	101/115 (87.9%)
Previous hysteroscopic treatments	
Polipectomy	15/115 (13.1%)
Myomectomy	9/115 (7.8%)
None	91/115 (79.1%)
Previous antibiotics therapy	
Yes	25/115 (21.7%)
No	90/115 (78.3%)

D&C, dilation and curettage; PID, pelvic inflammatory disease

No analgesics or anesthetics were administered during or after the hysteroscopic procedure, because it was performed in ambulatory outpatient setting without cervical dilatation. Through direct identification of endometrial areas suggestive of CE, targeted endometrial biopsies were collected [9–11] and all samples were examined by hematoxylin and eosin (H&E) staining. All the pathology slides of hysteroscopic biopsy were reviewed by two pathologists with great experience in endometrial pathology. Disagreements were resolved by discussion at a two-headed microscope. If an agreement was not obtained, a senior pathologist was consulted. The exclusion criteria were as follows: the presence of endometrial hyperplasia of any type, any antibiotic treatment for any o acute (< 15 days) infection and current pregnancy.

### Statistical analysis

Quantitative variables were expressed as means and standard deviations or medians as appropriate while absolute and relative frequencies were used for qualitative ones. The sensitivity, specificity, negative predictive value (NPV), positive predictive value (PPV), and accuracy for both techniques were calculated.

Cohen’s kappa index was used to evaluate the agreement between the obtained data. The kappa coefficient is a statistical measure that defines the observed consistency between categorical variants (pathologic results vs hysteroscopic findings), adjusted to compensate for the chance factor. For the determination of Cohen's kappa (κ), the following formula was applied: *κ* = (observed agreement [Po] − expected agreement [Pe])/(1 − expected agreement [Pe]). In particular, its use allows to determine the extent of the agreement, defining it as absent (< 0), scarce (0–0.19), modest (0.2–0.39), discreet (0.40–0.59), good (0.60–0.79), excellent (0.80–1).

## Results

Out of the 48 positive endometritis obtained from the histological report, hysteroscopies detected 31 real positive results and 17 false positives. Furthermore, of 67 women who had negative results according to the pathological examination, hysteroscopies detected 20 real positives, which means that the rate of false positives was of 29%. The sensitivity was 64% while the specificity 70%. The positive and negative predictive values were 60.8% and 73.4%, respectively, whereas the accuracy rate calculated was 67.8% (Tables 2 and 3).

**Table 2:** Presence/absence of chronic endometritis at pathology compared to hysteroscopic signs

	Endometritis at histopathology	No endometritis at histopathology	Total
Hysteroscopic endometritis signs	31	20	51
No hysteroscopic endometritis signs	17	47	64
Total	48	67	85

**Table 3:** Rates of sensitivity, specificity, positive predictive value (PPV) and negative predictive value (NPV), diagnostic accuracy and K Cohen among hysteroscopy and histopathology in the detection of chronic endometritis

	Hysteroscopy	Histopathology
Sensitivity % CI	64.6 (49.5–77.8)	60.8 (46.–74.2)
Specificity % CI	70 (57.7–80.7)	73.4 (60.9–83.7)
PPV % CI	60.8 (46.1–74.2)	64.6 (49.5–77.8)
NPV % CI	73.4 (73.4–60.9)	70.1 (57.7–80.7)
Diagnostic accuracy %	67.8	
K Cohen	0.34	

In addition, the result of the κ coefficient was 0.34, which is currently consistent with the agreement rate reported in literature so far (Table 3) [12, 13]. Based on the current literature, this discrepancy is considered a modest agreement.

Of the 115 selected hysteroscopies, 51 were diagnosed as CE by hysteroscopy, 20 showed signs of diffused or focal hyperemia, 1 hemorrhagic spots, 4 micropolyps as well as hyperemia, and the remaining 26 had micropolyps. This means that almost 50% of hysteroscopies presented micropolyps as a hysteroscopic sign. Dividing hysteroscopic signs in subgroups as seen in Table 4, our results showed consistency between histological and hysteroscopic results of 75% in presence of diffused or focal hyperemia, 53% in presence of just micropolyps and 100% in presence of hyperemia and micropolyps.

**Table 4:** Hysteroscopic signs in case of chronic endometritis (CE)

Hysteroscopic signs (subgroups)	Chronic endometritis (CE)		Total
	Presence	Absence	
Hyperemia (diffused or focal)	15	5	20
Hyperemia and micropolyps	5	0	5
Micropolyps	14	12	26

## Discussion

CE is a complex condition that requires a multidisciplinary approach. The role played by the immune system in endometritis is crucial. The cells of the immune system have been found in samples of CE: the main change lies within the alteration of the endometrial receptivity, caused by the migration of B lymphocytes from the basal layer to the glandular lumen, which implies the expression of multiple proinflammatory cytokines and adhesion molecules like E-selectin. Furthermore, it is well-known that in a healthy woman, during the second phase of the menstrual cycle and at the onset of pregnancy, there is an increase in NK cell population, which means that it is not uncommon to find it missing or diminished in women diagnosed with CE [4]. This, in addition to an increase in other apoptosis-regulating molecules (BCL2, BAX and Ki-67), promotes an unfavorable environment for correct implantation of fertilized oocyte in the endometrium.

Regarding the microbiological etiology of CE, there is not a unanimous consensus on which is the most frequent pathogen causing CE [5]. The microorganisms most involved and discussed in the literature are *Escherichia coli, Corynebacterium, Enterococcus faecalis, Staphyloccus, Kleibsella pneumoniae*, and *Mycoplasma* species; in addition, also genital pathogens associated with sexually transmitted infections such as *Chlamydia trachomatis, Ureaplasma urealyticum,* and *Neisseria gonorrhoeae* seems to be involved in CE. Thank new molecular methods for the detection and characterization of microorganisms in several fields of medicine, such as real-time (RT)-PCR, the diagnostic capacity to detect difficult-to-culture bacteria has been increasing, allowing both qualitative and quantitative results in an accurate and rapid manner [6]. In this regard, Moreno et al. have shown how molecular microbiology can detect bacterial pathogens causing CE and could be useful to guide a target therapy for this “tricky” endometrial condition, with diagnostic accuracy of 76.92% when matched to the classic diagnostic methods represented by histology, microbial cultures and hysteroscopy [7].

Regarding antibiotic treatment, several studies have demonstrated its efficacy, although it is still debated [12, 13]. In 2021, Cicinelli et al. demonstrated the superiority of antibiotic therapy compared with no treatment for CE cure (81.25% vs 6.25%), making a direct comparison with untreated controls, never performed until that moment, as well as a germ-oriented antibiotic therapy in every single case with a specific treatment based on endometrial culture and antibiogram results [13].

Nowadays, histological diagnosis is the gold standard for CE. In addition to the evaluation of the endometrial tissue, it involves identifying plasmatic cells in the endometrial stroma. Although it may seem easy, there are various limitations when it comes to carrying out a histological analysis of CE: indeed, accurately identifying plasmatic cells is sometimes compromised by their similarities with stromal endometrial fibroblasts. Furthermore, you have to take into account that such plasmatic cells can also be found in the endometrium of healthy women, without any association with inflammatory conditions. In addition to the lack of consensus as to the number of plasmatic cells necessary to make a CE diagnosis, which varies from a single one to a minimum of five plasma cells, a significantly lower percentage of NK cells in CE patients has been found compared with control patients: indeed, the expression of CD56^+^ CD16^−^ and of CD56^bright^ CD16^−^, markers of NK cells, was significantly lower (47.8% ± 18.6 and 30.1% ± 20.5 vs 79.5% ± 3.9 and 67.3% ± 8.1, respectively; *p* < 0.01) when compared to unexplained infertile women without any sign of CE [14, 15].

**Fig. 1:**
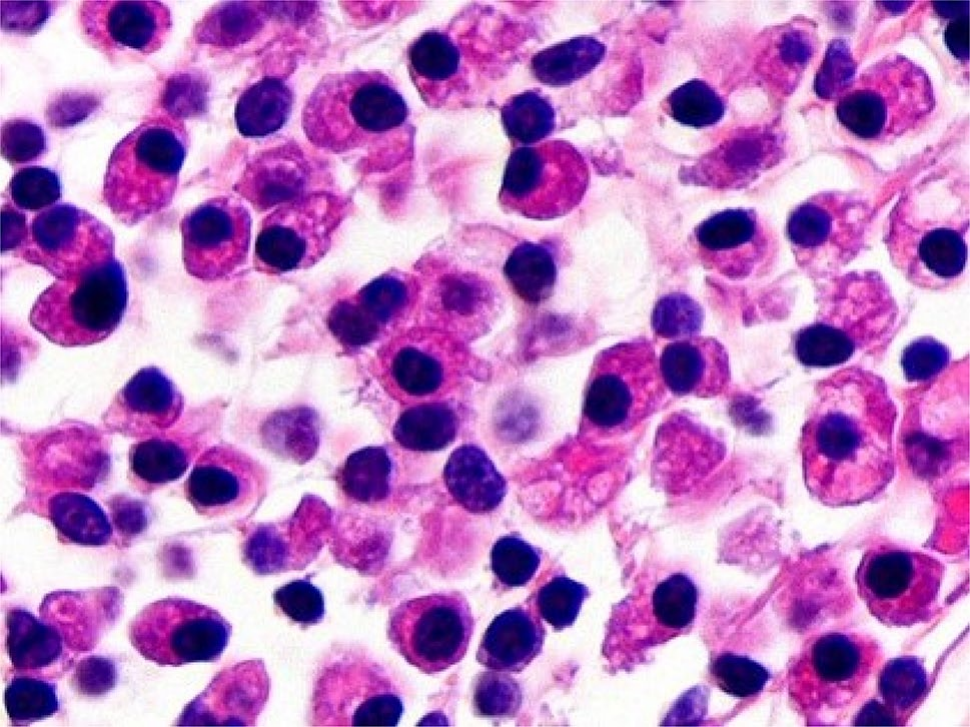
Hematoxylin–eosin staining of plasma cells in the endometrial stroma (× 400 pi)

**Fig. 2:**
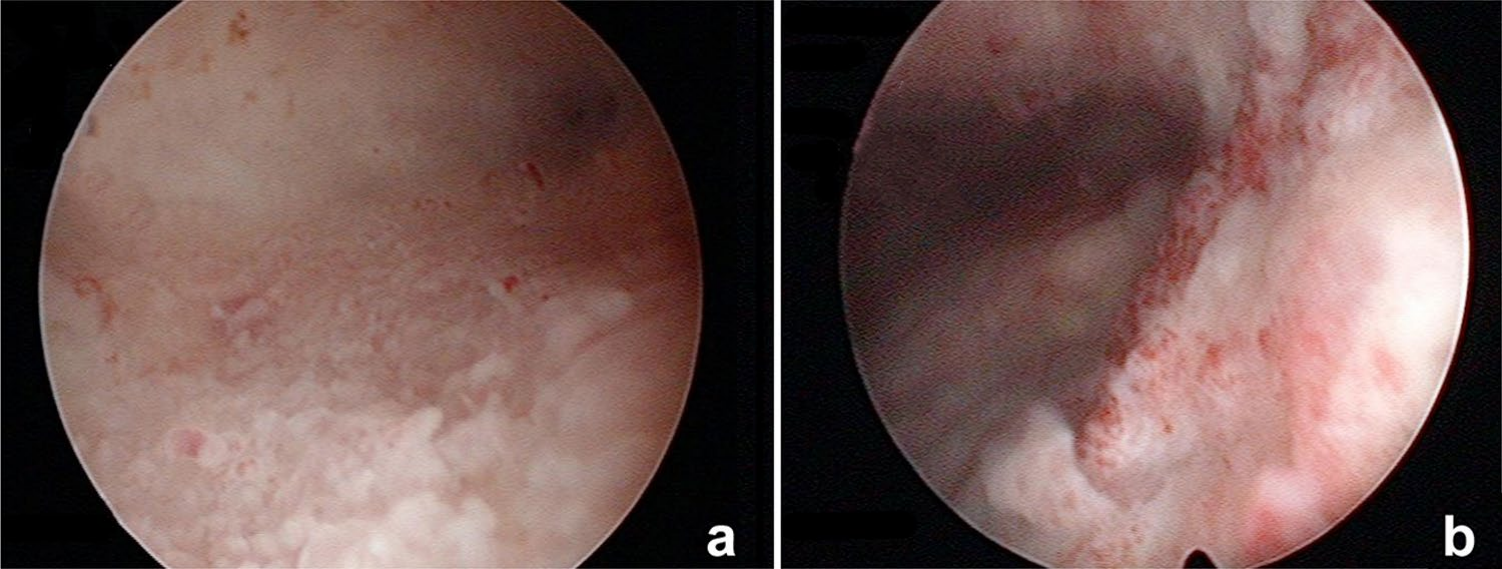
Typical hysteroscopic view of Chronic Endometritis (CE). **a** Diffuse micropolyps with a connective vascular axis; **b** hemorrhagic spots in a focal pale area in the endometrium

Normally, hematoxylin and eosin staining is used to identify such cells, which appear with basophilic cytoplasm and an elevated ratio nucleus/cytoplasm, as can be seen in Fig. 1. The detection of the plasmatic cells in the endometrial stroma has been improved thanks to the use of immunohistochemistry in the histological diagnosis of CE, by detecting the marker CD138, also known as Syndecan-1, a transmembrane (type I) heparan sulfate proteoglycan [14, 15]. Some authors suggest that the combination of both procedures is what offers a more reliable diagnosis [7, 8, 14, 15]. Both in the histological and hysteroscopic diagnoses, a certain degree of subjectivity on behalf of the pathologist and the endoscopist is undeniable, and therefore an increased interobserver variation. Nevertheless, recent studies aimed at trying to unify diagnostic criteria by focusing on hysteroscopic signs of CE, as described by Cicinelli et al. in 2019 who tried to develop a diagnostic consensus for CE, and such signs include micropolyps, focal hyperemia, strawberry endometrium, hemorrhagic spots and endometrial edema [5]. Regarding endometrial polyps, Nomiyama et al. recently described how they are associated with chronic endometritis in infertility patients in presence of an increased count of plasma cells [16]. A hysteroscopic view of CE in one of our patients is reported in Fig. 2.

As shown in Table 4, our results are consistent with the scoring-based system recently published by Liu et al., in which a minimum of 2 points is necessary in order to diagnose the presence of CE, and the presence of hyperemia by itself counts for 4 points (the highest) while the presence of micropolyps counts 1 point as an isolated hysteroscopic sign [8]. Thus, the presence of isolated micropolyps, according to this study, would not be sufficient to establish a positive hysteroscopic diagnosis of CE. On the other hand, less recent studies point to the hysteroscopic presence of micropolyps as a predictive sign of CE and as a trustworthy sign of inflammation [17, 18]. Based on what stated so far, the interest that CE is garnering nowadays in the world of female reproduction is triggering an increase in the number of published papers on the topic by many research groups throughout the world, in which a variety of results regarding the data on the hysteroscopy compared to histology has been shown. As with several endometrial diseases, including polyps, atypical hyperplasia and carcinoma, hysteroscopy is undoubtedly a diagnostic tool that proves very helpful and developing standardized criteria for the diagnosis of CE could in long run eliminate the discrepancies due to single operator [8, 15, 19–21]. One of the great aspects of hysteroscopy is the outpatient setting that safely allows to do an endometrial biopsy with no need for anesthesia or (in selected cases) just blunt analgesia [22].

Nevertheless, a histological confirmation should also be carried out in order to properly formulate an antibiotic treatment or any possible treatment, aimed at improving symptoms when present or reproductive outcome. Therefore, it would be desirable to develop new diagnostic methods which improve on the limitations of histological detection of plasmatic cells in the endometrial stroma [8, 23, 24]. The use of molecular analysis could improve the diagnosis of CE: about that, as mentioned above, Moreno et al. had promising results by amplifying and sequencing through Next Generation Sequencing (NGS) the gene 16S ribosomial RNA (rRNA), present in bacteria most frequently found in cases of CE, and confirming the detection of bacterial DNA in 12/13 endometrial samples [7].

Our study has some limitations, including the retrospective nature and the small sample size, which may be one of the factors that led to a modest agreement between hysteroscopy and final histology. Nevertheless, it has been conducted following a precise methodological rigour and including hysteroscopic and pathological evaluation by experts in the field, reducing inter-individual variability.

## Conclusions

Given the high estimated prevalence of CE and its relationship to an unfavorable reproductive outcome, it is paramount to establish and implement unified criteria among experts and non-experts to accurately diagnose it. To date, it seems clear that hysteroscopy or histological examination alone cannot allow in all cases the diagnosis of CE. Our study seeks to describe the results perceived at our Center during a limited length of time, but it could surely benefit from a greater pool of data which would allow other compelling statistical measures. New lines of research based on molecular biology are opening interesting new pathways and will undoubtedly allow for improvement when diagnosing this condition.

## Data Availability

Data are available upon request by the Corresponding Author.
