# Correction: Hysteroscopic versus histopathological agreement in the diagnosis of chronic endometritis: results from a retrospective observational study

**DOI:** 10.1007/s00404-023-07278-0

**Published:** 2023-11-16

**Authors:** Belén Almoguera Pérez-Cejuela, Salvatore Giovanni Vitale, Tirso Pérez-Medina, Mar Rios-Vallejo, Luigi Della Corte, Ana Royuela Vicente, Stefano Angioni, Laura Calles-Sastre

**Affiliations:** 1 Gynecology and Obstetrics Department, Puerta de Hierro Hospital, Autónoma University of Madrid, 28223 Majadahonda, Madrid Spain; 2 Division of Gynecology and Obstetrics, Department of Surgical Sciences, University of Cagliari, 09124 Cagliari, Italy; 3 Department of Neuroscience, Reproductive Sciences and Dentistry, School of Medicine, University of Naples Federico II, 80131 Naples, Italy

**Correction: Archives of Gynecology and Obstetrics (2023) 308:1817–1822** 10.1007/s00404-023-07163-w

In this article Laura Calles-Sastre was incorrectly denoted as the corresponding author but it should have been Tirso Pérez-Medina.

The original article has been corrected.

